# Nisin E Is a Novel Nisin Variant Produced by Multiple *Streptococcus equinus* Strains

**DOI:** 10.3390/microorganisms11020427

**Published:** 2023-02-08

**Authors:** Ivan Sugrue, Daragh Hill, Paula M. O’Connor, Li Day, Catherine Stanton, Colin Hill, R. Paul Ross

**Affiliations:** 1 APC Microbiome Ireland, Biosciences Institute, University College Cork, T12 YT20 Cork, Ireland; 2 Teagasc Food Research Centre, Moorepark, Fermoy, Co., P61 C996 Cork, Ireland; 3 Grasslands Research Centre, Te Ohu Rangahau Kai, AgResearch, Palmerston North 4410, New Zealand

**Keywords:** nisin, streptococcus equinus, bacteriocin, lantibiotic, antimicrobial peptide, biotherapeutic

## Abstract

Nisin A, the prototypical lantibiotic, is an antimicrobial peptide currently utilised as a food preservative, with potential for therapeutic applications. Here, we describe nisin E, a novel nisin variant produced by two *Streptococcus equinus* strains, APC4007 and APC4008, isolated from sheep milk. Shotgun whole genome sequencing and analysis revealed biosynthetic gene clusters similar to nisin U, with a unique rearrangement of the core peptide encoding gene within the cluster. The 3100.8 Da peptide by MALDI-TOF mass spectrometry, is 75% identical to nisin A, with 10 differences, including 2 deletions: Ser29 and Ile30, and 8 substitutions: Ile4Lys, Gly18Thr, Asn20Pro, Met21Ile, His27Gly, Val32Phe, Ser33Gly, and Lys34Asn. Nisin E producing strains inhibited species of *Lactobacillus*, *Bacillus*, and *Clostridiodes* and were immune to nisin U. Sequence alignment identified putative promoter sequences across the nisin producer genera, allowing for the prediction of genes in *Streptococcus* to be potentially regulated by nisin. *S. equinus* pangenome BLAST analyses detected 6 nisin E operons across 44 publicly available genomes. An additional 20 genomes contained a subset of nisin E transport/immunity and regulatory genes (*nseFEGRK*), without adjacent peptide production genes. These genes suggest that nisin E response mechanisms, distinct from the canonical nisin immunity and resistance operons, are widespread across the *S. equinus* species. The discovery of this new nisin variant and its immunity determinants in *S. equinus* suggests a central role for nisin in the competitive nature of the species.

## 1. Introduction

Bacterial antimicrobial resistance (AMR) caused an estimated 1.27 million deaths in 2019, which is predicted to rise to 10 million annually by the year 2050, according to the World Health Organisation [1,2]. Novel antimicrobials that can be utilised to combat AMR pathogens are increasingly in demand. Produced by bacteria, bacteriocins are a heterogeneous group of ribosomally-synthesised peptides and post-translationally modified peptides (RiPPs) with antimicrobial activity [3,4]. They are small (<10 kDa), with either broad or narrow inhibition spectra and are classified into groups and subgroups according to their structure [5,6]. Class I bacteriocins are post-translationally modified and include lantibiotics, peptides in which serine and threonine residues are dehydrated enzymatically to 2,3-didehydroalanine (Dha) and 2,3-didehydrobutyrine (Dhb), which in turn form lanthionine and methyllanthionine thioether bridges with neighbouring cysteines [7]. Bacteriocins could be an alternative or adjunct to antibiotics, for certain applications.

Nisin is the prototypical lantibiotic, first described in 1928 as an ‘inhibiting effect’ produced by *Lactococcus lactis* on *Lactobacillus bulgaricus* [8]. The 34-amino acid lantibiotic has been studied extensively, was granted generally recognised as safe (GRAS) status in 1988, and was approved for use as a food ingredient by EFSA under the code E234 [9]. The structure of nisin A produced by *L. lactis* includes five rings, a three-residue flexible ‘hinge’ region, and a five-residue cationic tail that interacts with cell membranes and is crucial for activity [10]. Nisin inhibits a broad range of Gram-positive bacterial genera, including many clinically relevant pathogens, biofilms, and sporeformers [11,12,13]. Nisin has also been subject to extensive site-directed mutagenesis, which has identified functionally critical regions in which amino acid substitutions increase or decrease peptide activity against particular organisms [14,15,16].

More than ten natural variants of nisin have been identified since the discovery of nisin A. Nisin Z is produced by *L. lactis* and contains a single amino acid substitution (His27Asn) [17]. Nisin F and Q are also produced by members of the genus *Lactococcus* and exhibit only subtle differences in sequence [18,19]. More recently, distantly related nisin J, nisin O (O123, and O4), and kunkecin A were identified from human skin, gut, and honeybee microbiota, and are produced by *Staphylococcus capitis*, *Blautia obeum*, and *Apilactobacillus kunkeei* FF30-6, respectively [20,21,22]. The genus *Streptococcus* has been a rich source of natural nisin variants, and species of diverse origin produce nisin variants U/U2, H, P, and most recently, G [23,24,25,26,27].

Nisin expression is autoregulated by a two-component histidine kinase/response regulator quorum sensing system (*nisRK*) encoded within the nisin gene cluster [28]. Cells sensing extracellular mature nisin activate the expression of lantibiotic immunity and production machinery from two NisRK regulated promoters [29]. The NisRK two-component system is also exploited by a separate nisin resistance cassette identified in *Streptococcus* spp., which confers resistance to nisin through proteolytic cleavage and peptide export [30].

In this study, we aimed to isolate and characterise novel bacteriocin-producing lactic acid bacteria. Upon identification of two nisin variant-producing *Streptococcus equinus* strains, we sought to establish their novelty relative to other nisin producers through genetic comparison and to determine the prevalence of this nisin variant in the species. Comparative analysis of nisin variant gene clusters revealed specific regulatory elements of *Streptococcus equinus* and other *Streptococcus* spp. highlighting conservation and diversity across nisin expression regulation systems.

## 2. Materials and Methods

### 2.1. Strain Isolation, Bacteriocin Activity Screening, and Speciation of Isolates

In total, 112 samples, consisting mainly of raw ovine, bovine, and caprine milk (Supplementary Table S1) were spread on several media for the isolation of putative lactic acid bacteria, as described previously ([31]). Briefly, samples were streaked or serially diluted and plated on *Streptococcus thermophilus* selective agar; M17 agar with 10% lactose; de Man, Rogosa, and Sharpe (MRS) agar containing 30 μg·mL^−1^ vancomycin, MRS adjusted to pH 5.4; *Lactobacillus* selective agar (LBS); and transgalactosylated oligosaccharide (TOS) agar, supplemented with 50 μg·mL^−1^ lithium mupirocin, and incubated for 24 to 72 h at 42 °C, 30 °C, and 37 °C, aerobically, and 42 °C, 30 °C, and 37 °C, anaerobically, respectively. All isolates were screened for bacteriocin production by overlaying with sloppy MRS agar (0.75% wt/vol agar), pre-tempered to 50 °C and seeded with 0.25% (vol/vol) of an overnight *Lactobacillus delbrueckii* ssp. *bulgaricus* LMG6901 culture. Colonies producing distinct zones of inhibition were triple-streaked for purity and cultured in broth overnight to produce a cell-free supernatant (CFS) for subsequent well diffusion assays. Overnight cultures were centrifuged at 16,000× *g* for 3 min and the resulting supernatant was filtered through a 0.2 µm filter (Sarstedt, Wexford, Ireland), yielding CFS. For well diffusion assays, 20 mL volumes of sloppy MRS agar seeded with *L. bulgaricus* LMG6901 were poured into petri dishes and allowed to set. Six-millimeter wells were bored in the agar using glass Pasteur pipettes, into which 50 µL CFS was added. Plates were examined for zones of inhibition following overnight incubation. Supernatants producing zones of inhibition (active supernatant) were treated with 20 mg·mL^−1^ proteinase K (Merck) for 3 h to digest proteinaceous compounds, and the well diffusion assays were repeated. Loss of activity denoted a proteinaceous compound. Potential bacteriocin producers were subject to MALDI-TOF mass spectrometry, as previously described ([31]).

### 2.2. Strain Speciation and Genomic Comparison

Strains of interest were subject to genomic DNA extraction using the GenElute Bacterial Genomic DNA kit (Merck, Wicklow, Ireland) and 16s rRNA gene sequencing (Genewiz, Leipzig, Germany) with the 27F (5′-AGAGTTTGATCCTGGCTCAG-3′), U1492R (5′-GGTTACCTTGTTACGACTT-3′) universal primers. Genomic DNA was quantified with a Qubit 2.0 fluorometer and prepared for sequencing with the Illumina Nextera XT kit, according to manufacturer protocols. Sequencing was performed using the Illumina MiSeq platform with paired-end 2 × 300-bp reads using the Teagasc Sequencing Centre, (Teagasc Moorepark Food Research Centre, Fermoy, Cork, Ireland). Assemblies were performed *de novo* with SPADES (v 3.0.0) [32]. Contigs were aligned to reference genomes using Mauve (version 20150226, build 10), and annotation was performed using RAST (version 2.0) [33,34]. Any further annotation was performed using the Artemis genome browser (version 16.0) [35]. Average nucleotide identity was calculated relative to related species genomes using OrthoANI (version 0.93.1), with publicly available *Streptococcus* spp. reference genomes from NCBI [36]. Draft genomes were subject to bacteriocin gene cluster prediction with BAGEL4 [37]. Amino acid sequences of encoded bacteriocin peptides were aligned to homologues using MUSCLE and visualised using Jalview [38,39]. Percent identity matrices were generated using Clustal Omega [40]. A dendrogram of aligned peptide sequences was generated using SimplePhylogeny [41] and visualised using iTOL [42]. *Streptococcus* genomes were examined for genomic differences using the Mauve genome aligner, and Easyfig (version 2.1) [43]. Following genome sequencing, a 69 bp contig boundary gap was present within the *nseB* gene encoded by *S. equinus* APC4008. To close the gap and confirm the contiguous nature of the gene cluster, a PCR was performed with KOD Hot Start master mix (Merck) using the primers nisE_F (5′ CTGCCCGTTGGAGGTTAAGT 3′) and nisE_R (5′ ACAGTGTGCTTAGGACAAACA 3′), with a denaturation step of 96 °C for 2 min and 30 cycles of 96 °C for 15 s, 55 °C for 15 s, 72 °C for 20 s, and a final extension of 72 °C for 10 min. The single resulting 794 bp product was purified using a GenElute PCR purification kit (Merck) and sent for sequencing (Genewiz, Leipzig, Germany). The sequence was examined for quality using Chromas (version 2.6.6), then aligned to the corresponding region within *S. equinus* APC4007 and 4008, using Clustal Omega and Jalview.

### 2.3. Streptococcus Equinus Pangenome Analysis

Publicly available *S. equinus* sequences were acquired from ncbi.nlm.nih.gov/datasets, accessed on 10 March 2022 (Supplementary Table S2). Local BLAST+ executables were downloaded (ftp.ncbi.nlm.nih.gov/blast/executables/blast+/LATEST/, accessed on 10 March 2022) and used to construct local nucleotide and protein databases from *S. equinus* genomes. The nucleotide database was interrogated using the 13,339 bp nucleotide sequence of the nisin E operon from *S. equinus* APC4007. The protein database was constructed and interrogated with *Streptococcus* sp. Nisin resistance and nisin immunity protein sequences were acquired from UniprotKB [44] (Supplementary Tables S3 and S4).

### 2.4. Nisin Variant Cross-Immunity Assay

*L. lactis* ATCC11454, *L. lactis* NZ9800 pCI372-nisA, *L. lactis* NZ9800 pCI372-nisZ, *L. lactis* NZ9800 pCI372-nisF, *L. lactis* NZ9800 pCI372-nisQ, *Staphylococcus capitis* APC2923, *S. uberis* 42, *S. equinus* APC4007, *S. equinus* APC4008, *S. hyointestinalis* DPC6484, and *S. agalactiae* DPC7040 (nisin A, A, Z, F, Q, J, U, E, E, H, and P producers, respectively) were cultivated in the appropriate broth medium from fresh streak overnight (Table 1). The strains were stocked in a 96-well plate with glycerol to a final concentration of 20% (vol/vol). Using a 96-pin replicator, the nisin producers were stamped on M17 containing lactose, M17 containing glucose, and BHI agar plates. Following incubation overnight, the plates were subjected to agar overlay, as described above, with 50 mL sloppy agar inoculated either with a nisin producer or indicator species.

### 2.5. Promoter Prediction and Transcription Start Site Mapping

Nucleotide sequences containing nisin gene clusters were obtained (Supplementary Table S5) and used for promoter prediction by sequence alignment to known promoters in *L lactis* ssp. *lactis* [29]. Alignments were generated with Clustal Omega, and visualised with Jalview. Rho-independent terminators were predicted using ARNold [45], under default settings.

## 3. Results

### 3.1. Isolation of Two Bacteriocin-Producing Streptococcus equinus Strains

**Figure 1:**
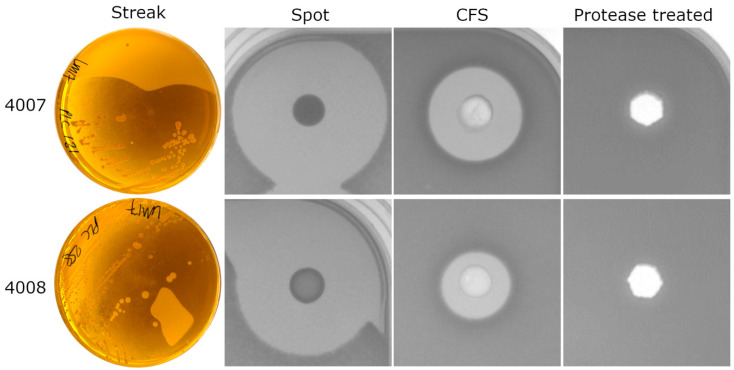
Zones of inhibition of *Lactobacillus delbrueckii* ssp. *bulgaricus* LMG6901 produced by *Streptococcus equinus* strains APC4007 (**top**) and APC4008 (**bottom**) in deferred antagonism assays, and well diffusion assay of cell free supernatant (CFS) and CFS treated with proteinase K.

*S. equinus* APC4007 and *S. equinus* APC4008 were initially isolated from separate sheep milk samples as small (1–3 mm diameter), round, convex, creamy-white, semi-translucent colonies. The colonies produced zones of inhibition against the acid-tolerant indicator species *Lactobacillus delbrueckii* ssp. *bulgaricus* LMG6901 in agar overlays of colonies and spots on the plates (Figure 1). *L. bulgaricus* LMG6901 was also inhibited by the pH-neutralised cell-free supernatant of the isolates in a well diffusion assay indicating export of a soluble antimicrobial compound. This activity was found to be sensitive to treatment with proteinase K, suggesting that the compound was proteinaceous in nature (Figure 1).

*S. equinus* APC4007 and *S. equinus* APC4008 were subject to 16s rRNA gene sequencing and identified as *Streptococcus* sp. with 97–99% identity to *Streptococcus lutetiensis, S. equinus*, and *Streptococcus infantarius*. *S. equinus* APC4007 and 4008 were subject to whole-genome shotgun sequencing to speciate and characterise the strains, resulting in two draft genomes consisting of ten (JANHMF000000000) and nine (JANHME000000000) contigs, respectively. Average nucleotide identities of both strains were calculated relative to complete genomes of *S. equinus* MDC1, *S. equinus* NCTC8140, *S. lutetiensis* NCTC13774, *S. infantarius* FDAARGOS 1019, and *S. gallolyticus* ssp. *gallolyticus* DSM 16831 (RefSeq accessions: GCF_014041875.1, GCF_900636465.1, GCF_900475675.1, GCF_016127275.1, and GCF_002000985.1, respectively) (Supplementary Figure S1). The two isolates shared the highest identity with *S. equinus* MDC1 and *S. equinus* NCTC8140 and thus, were designated as *S. equinus* species. 

### 3.2. Nisin E Is a Novel Variant Unique to Streptococcus equinus

**Figure 2:**
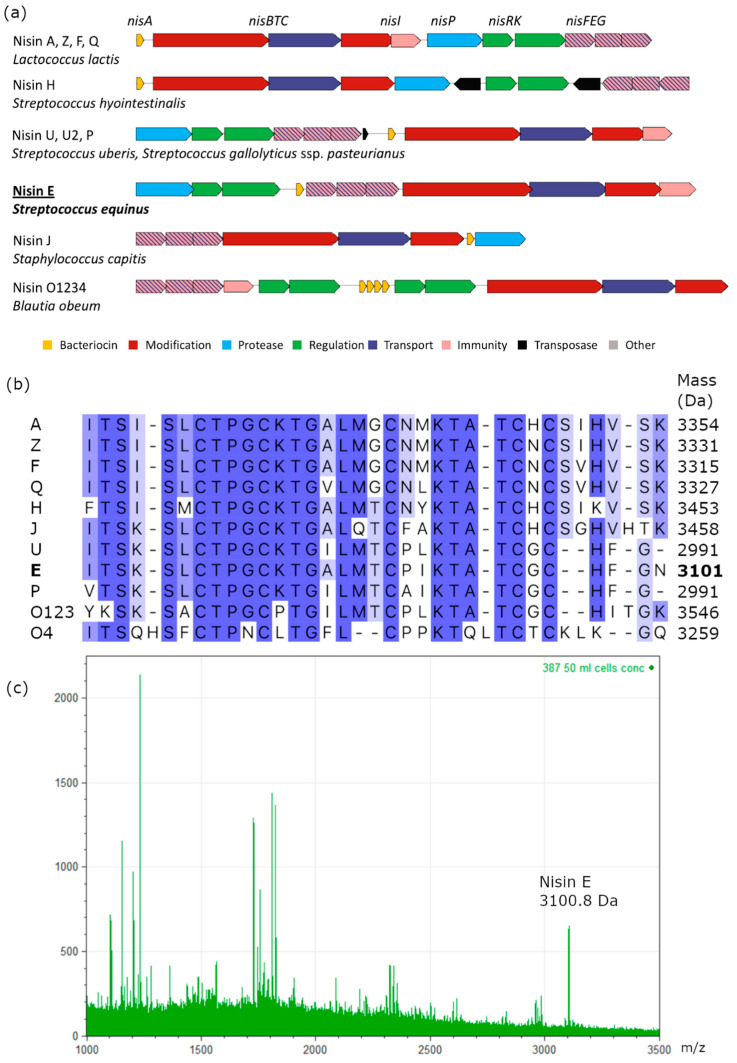
(**a**) Gene cluster structure comparison of natural nisin variants, including nisin E structure in *Streptococcus equinus*. (**b**) Amino acid sequence alignment of the nisin E propeptide to its related variants and nisin A; bold: predicted mass of nisin E. (**c**) MALDI-TOF colony mass spectra of *S. equinus* APC4007 displaying the nisin E mass at 3100.7 Da.

**Figure 3:**
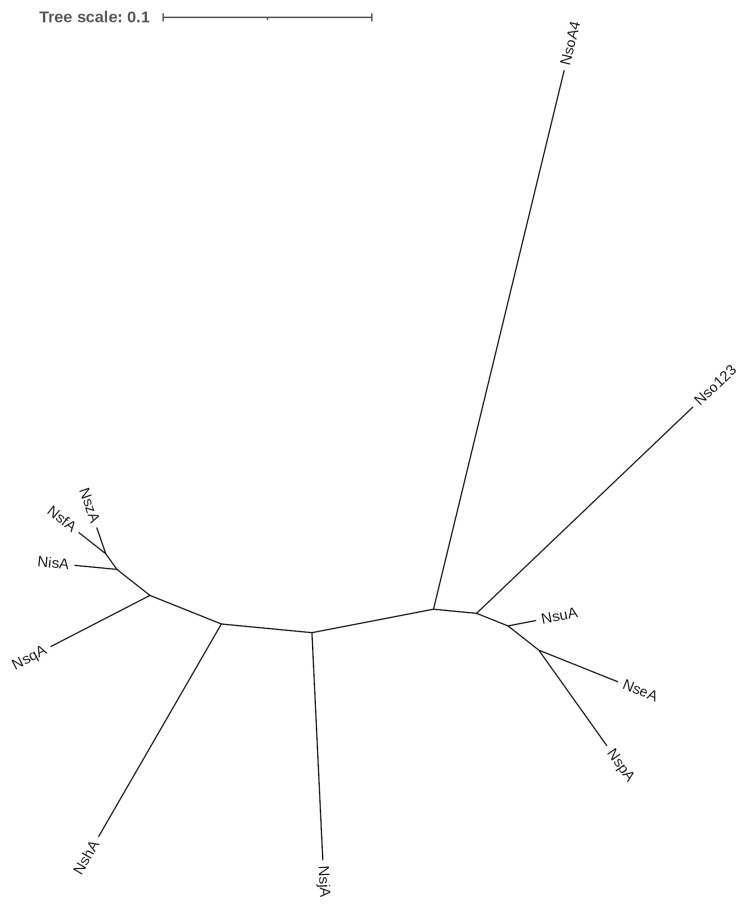
Dendrogram of nisin E in relation to other natural nisin variants. The tree scale represents branch length and indicates amino acid substitutions per position.

The genomes of *S. equinus* APC4007 and 4008 were found to encode highly similar nisin biosynthetic gene clusters (Figure 2a). The nucleotide region of the nisin production gene clusters was 99.86% identical between the two strains, containing 16 single nucleotide polymorphisms. The gene cluster organisation does not match any previously described nisin variant (Figure 2a). The gene clusters resemble the nisin U gene cluster, with genes corresponding to *nsuBTCI (nseBTCI)* located downstream of *nsuPRKFEG (nsePRKFEG)* (relative to nisin A in *L. lactis*). The position of the structural gene *nseA*, between *nseFEG* and *nsePRK*, is unlike the nisin A or U gene clusters (Figure 2a). Both strains encode a structural peptide, designated as nisin E, which shares 76.4% and 75% amino acid identity with the nisin A prepropeptide and the leaderless peptide, respectively (Figure 2b). Nisin E is 32 amino acids in length, containing 10 differences from nisin A; two deletions, Ser29 and Ile30, and eight substitutions, Ile4Lys, Gly18Thr, Asn20Pro, Met21Ile, His27Gly, Val32Phe, Ser33Gly, and Lys34Asn. The cleaved peptide is similar to nisin U, sharing 93.6% identity, two amino substitutions, Ile15Ala, Leu21Ile, and one additional C-terminal Asn32 residue (Figure 2b). The unmodified mass of the nisin E peptide is predicted to be 3245.9 Da and 3101 Da, following eight dehydrations. Mass spectrometry detected a mass of 3100.8 Da, corresponding to the mature peptide produced by *S. equinus* APC4007 and 4008 (Figure 2c). A dendrogram of peptide relatedness clustered nisin E with other *Streptococcus* derived nisins, U and P, in addition to O1 and O4 from *Blautia obeum* (Figure 3). *S. hyointestinalis* DPC6484′s nisin H clustered more closely with the lactococcal nisins A, Z, F, and Q.

### 3.3. A Predicted Streptococcus-Specific Promoter for Expression of nisP

**Figure 4:**
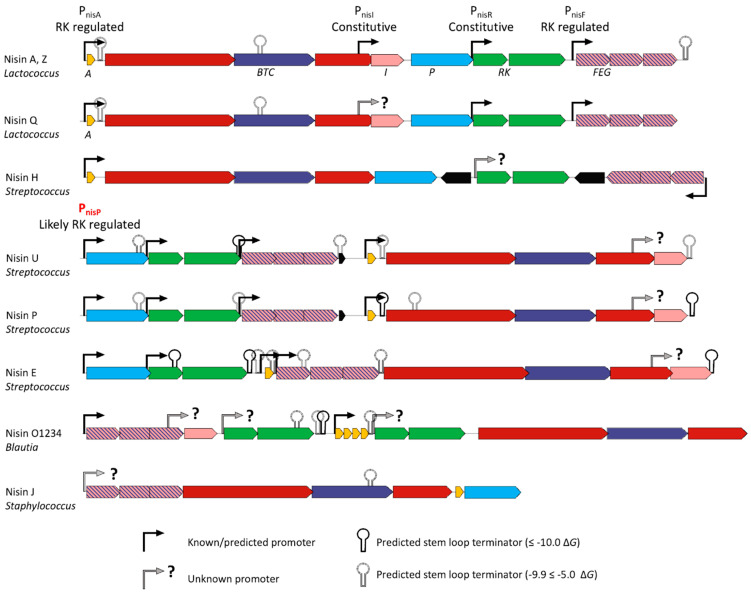
Predicted promoters of nisin variant production operons. Absent promoter sequences are denoted by arrows with question marks. Rho-independent transcription terminators of the forward strand with a free Gibbs energy (Δ*G*) lower than −5.0 kcal/mol are denoted by stem loop structures, predicted by ARNold.

Given the novel layout of the nisin E gene cluster, we sought to characterise the promoters responsible for nisin E expression through multiple sequence alignments of nucleotide regions upstream of *nisA*, *nisF, nisR*, and *nisI*-type genes of the A, Z, Q, H, P, U, E, J, and O type. These alignments revealed some conservation of promoter sequences across genera (Figure 4, Supplementary Figures S2–S4). Rho-independent terminator prediction software identified 30 transcription terminators, with a Gibbs free energy (Δ*G*) stronger than −5.0 kcal/mole across the nisin variant gene clusters (Figure 4, Supplementary Table S6). The nisin E gene cluster contained the most predicted terminators (7), followed by U (5), P (5), O (4), A (3), Z (3), Q (2), and J (1). None were predicted within the nisin H gene cluster. Seven putative terminator sequences were predicted to have a Δ*G* stronger than −10.0 kcal/mole, six of which are present in streptococcal gene clusters. The nisin E gene cluster contains three strong terminators, one within the *nseR* open reading frame, one immediately following *nseK* upstream of *nseA*, and a third following *nseI.* Nisin A, Z, Q, P, U, E, and O gene clusters contain predicted terminators of various strengths, immediately following the core peptide encoding genes (Figure 4).

The predicted promoter upstream of the *nisR* homologues in the Z and Q gene clusters (P_nszR_, and P_nsqR_) are homologous to *L. lactis* ssp. *lactis* (P_nisR_), which we designate *Lactococcus* type (L type) (Supplementary Figure S3). *S. equinus* APC4007, APC4008, *S. uberis* 42, *S. agalactiae* DPC7040, and *Blautia obeum* A2-162 share a distinct conserved predicted *nisR* promoter structure, which we designate as *Streptococcus* type (S type), that encodes a −35 and −10 nucleotide sequence of TGCACA and TATTAC, respectively, separated by 15 nucleotides (Supplementary Figure S3). *S. hyointestinalis* DPC6848 (nisin H) does not share the conserved −35 or −10 of either type upstream of *nshR*. The nisin O gene cluster encodes two copies of *nisRK* homologs, neither of which have nucleotide sequences that are similar to the predicted *nisR* promoters in *Streptococcus* or *Lactococcus* spp. (Supplementary Figure S3). Alignment of the 400 bp upstream of *nisI* and its homologues identified no obvious promoter elements conserved across species (Supplementary Figure S4).

The promoter responsible for the expression of the core peptide in *L. Lactis* (P_nisA_) is somewhat conserved in the Z, Q, H, O, U, E, and O operons with a −35 and −10 consensus of CTGAAC and TACAAT, respectively, with a non-canonical spacer of 20 nucleotides (Supplementary Data S9). The non-canonical −35 sequence is part of a conserved TCT-N8-TCT repeat, which is largely conserved across the NisRK regulated promoters, and is also present 54 bp upstream of the −35 in the nisin A and Z operons (Supplementary Figure S2). *Staphylococcus capitis* APC2923 (nisin J producer) does not encode a similar conserved promoter and is lacking an apparent −10 signal. The promoter responsible for *nisF* and its downstream genes is also conserved across the nisin operons, with a consensus of TGAACA and TATACT for the −35 and −10 regions, respectively, and a spacer length measuring 19 nucleotides (Supplementary Figure S2). Alignments of the DNA sequence upstream of the serine peptidase encoding gene (*nisP*) of *S. equinus* (nisin E), *S. agalactiae* (nisin P)*,* and *S. uberis* (nisin U) revealed homology with the NisRK regulated promoters described above (Supplementary Figure S2). Upstream of *nseP* in *S. equinus* APC4007 and APC4008, respectively, a conserved sequence of CTGAAC and TAAAAT is present, and these sequences are nearly identical to the *nisA* consensus sequences of CTGAAC and TACAAT (Supplementary Figure S2).

### 3.4. Spectrum of Inhibition of Nisin E Producers and Cross-Immunity to Other Nisin Producers

The spectrum of inhibition of *S. equinus* APC4007 and 4008 was determined by deferred antagonism assay against 40 Grampositive indicators. Both strains inhibited the growth of seven indicator species tested (Table 2). Strong inhibition was observed against *Lactobacillus bulgaricus* LMG6901, *Lactobacillus delbrueckii* ssp. *lactis* DPC5387, and *Bacillus firmis* DPC6349. *Lactobacillus helveticus* DPC5358, *Ligilactobacillus salivarius* DPC6502, *Clostridioides difficile* DPC6534, *Clostridium sporogenes* LMG10143, *S. intermedius* DSM20373, and *L. lactis* HP were weakly inhibited (Table 2). No inhibition was observed against other *Bacillus* and *Staphylococcus* spp., *Enterococcus* spp., *Listeria* spp., or other streptococci (Table 2).

**Figure 5:**
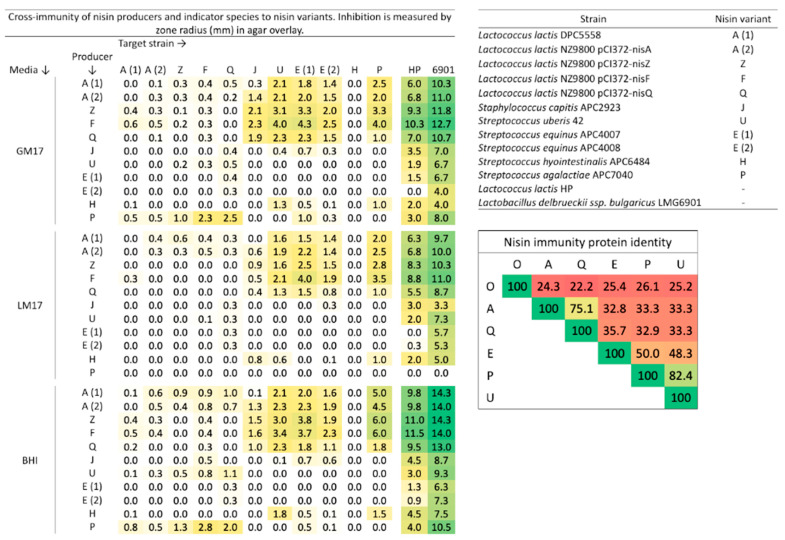
Cross immunity of natural nisin variants with nisin E producers on different media, determined by deferred antagonism assay. Inhibition is displayed as the zone radius (mm). Inset: amino acid percent identity matrix of the nisin immunity protein (NisI) and its homologues.

The cross-immunity of nisin E producers against other nisin producers was determined by a deferred antagonism assay on different growth media. *S. equinus* APC4007 and APC4008 were inhibited by nisin A, Z, F, and Q producers, weakly inhibited by nisin J, H, and P producers (<1.0 mm zone radius), and not inhibited at all by the nisin U producer (Figure 5). Nisin E producers failed to inhibit any nisin producers except for *L. lactis* NZ9800 pCI372-nisQ, which was weakly inhibited on all media. Both *S. equinus* APC4007 and APC4008 were weakly active against *L. lactis* HP and were consistently more active against *L*. *bulgaricus* LMG6901 on each media type (Figure 5).

Nisin production from all strains was improved by growth on BHI agar, when compared with M17 media containing glucose or lactose, as was evidenced by increased zone sizes against the non-nisin-producing indicators, *L. lactis* HP and *Lactobacillus delbrueckii* ssp. *bulgaricus* LMG6901 (Figure 5). *S. agalactiae* APC7040 (nisin P) failed to inhibit any strain when cultured on M17 agar containing lactose, despite evident growth. *S. hyointestinalis* DPC6484 (nisin H) was not inhibited by any nisin variant producer on any media type (Figure 5).

### 3.5. Nisin E Immunity Genes Are Spread throughout the Streptococcus equinus Pangenome

**Figure 6:**
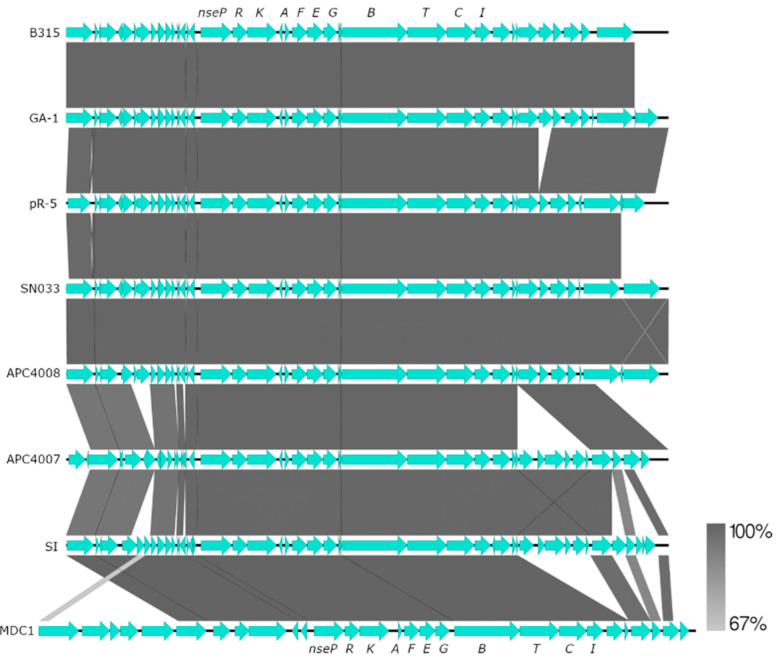
Multiple comparison of nisin E gene cluster (*nsePRKAFEGBTCI*) and its neighbouring genes detected in *Streptococcus equinus* genomes. Scale represents nucleotide identity.

**Figure 7:**
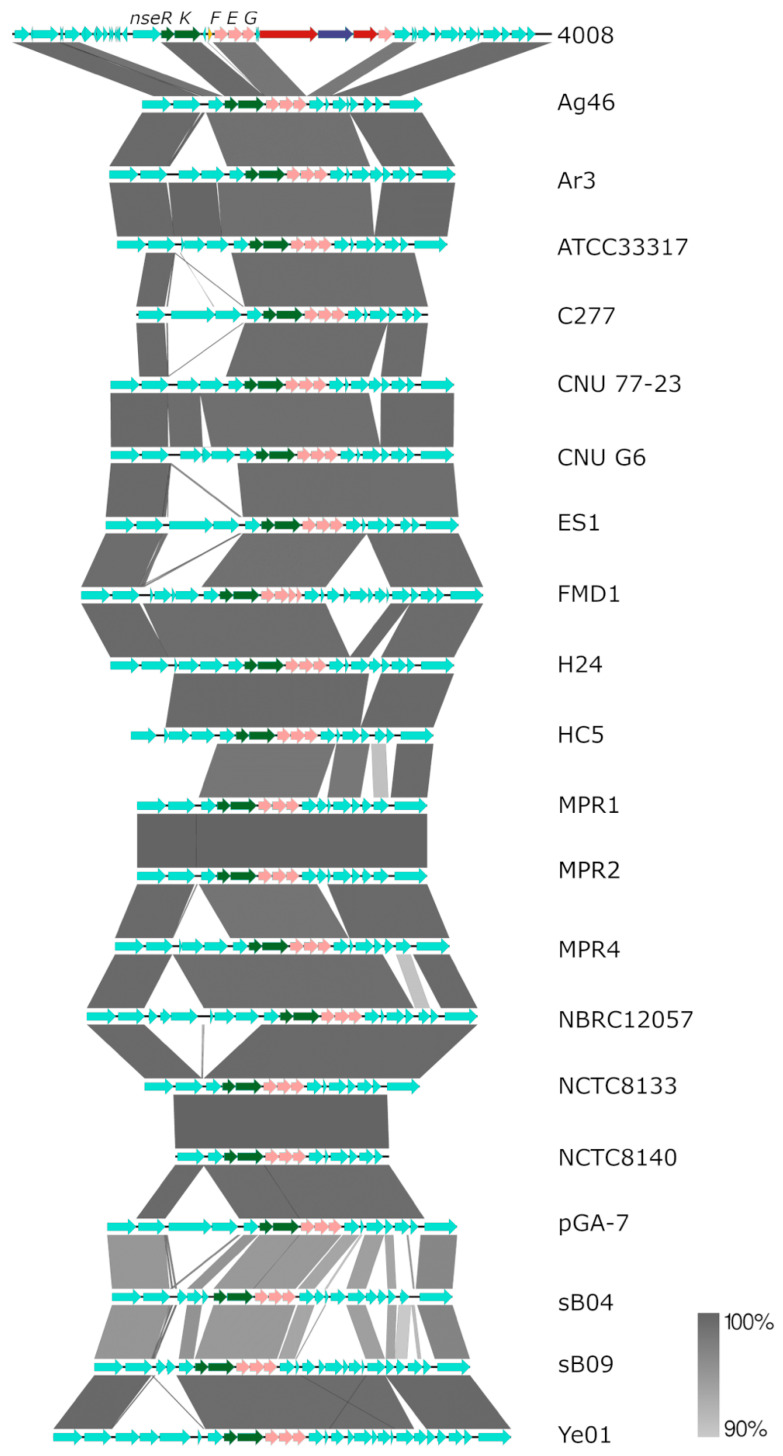
Multiple comparison of nisin E gene cluster and neighbouring open reading frames in *Streptococcus equinus* APC4008 with *nseRKFEG*, identified in publicly available *S. equinus* genomes. Scale represents nucleotide identity.

The *nsePRKAFEGBTCI* gene cluster, encoding nisin E production, was found in 6 of 44 publicly available *S. equinus* genomes (B315-G597, GA-1, SN033, pR-5, SI, MDC1), in addition to *S. equinus* APC 4007 and 4008 (Figure 6, Supplementary Table S7). Gene synteny is conserved within the production gene cluster across all the genomes, but differs approximately 10kb upstream and downstream of *nse* genes in both APC4007 and 4008 (Figure 6). A subset of 20/44 *S. equinus* genomes (45%) encode the nisin E histidine kinase/response regulator, and transport/immunity proteins (*nseRKFEG*) (Figure 7, Supplementary Table S7). These genes are present, without the corresponding nisin E production machinery. The encoded NseRKFEG proteins share a high level of amino acid identity with the corresponding proteins of the complete nisin E gene cluster, as opposed to homologous proteins encoded by nisin U (NsuRKFEG) or nisin A (NisRKFEG) (Supplementary Figure S5). A database of proteins extracted from the 44 public *S. equinus* genomes was searched for sequences homologous to the nisin resistance protein (Nsr), from which no significantly similar hits were identified. The same database was screened for the presence of nisin immunity protein (NisI) homologs, and only the immunity proteins encoded within the previously identified nisin E gene clusters were detected.

## 4. Discussion

We describe the production of a novel nisin E variant by two *S. equinus* strains, APC4007 and APC4008, that were isolated from unpasteurised sheep milk sampled from geographically separate locations in New Zealand; we then further describe the prevalence of this variant across the *S. equinus* pangenome using publicly available sequences. Nisin E described the following nisins: Z, U, F, Q, H, O1,2,3, and O4, P, J, G, and kunkecin A [17,18,19,20,21,22,23,24,25,26,27]. Streptococcal variants now compose 45% (U, H, P, G, E) of the natural nisin variants described, which may suggest an importance of their role in *Streptococcus* spp. competition and in niche colonisation or quorum sensing. Of the five streptococcal variants, two (P and E) are produced by members of the taxonomically complicated *S. bovis/S. equinus* complex (SBSEC) [25]. The SBSEC consists of seven species and subspecies which are mainly commensal bacteria that colonise gastrointestinal tracts of animals [46]. Species of the SBSEC are found in unpasteurized ruminant milk, likely via contamination of the teat skin and the teat canal. SBSEC members are implicated in infection and antimicrobial resistance, but are also used in food fermentation. Bacteriocin production by members of the SBSEC has been described previously, including bovicin HC5, bovicin HJ50, bovicin 255, macedocin, macedovicin, gallocin, and gallocin D [47,48,49,50,51,52,53]. SBSEC member bacteriocin production has been suggested as a mechanism that may support diverse niche colonisation, including opportunistic pathogenesis [52,53,54]. As such, nisin E may confer an advantage to strains of *S. equinus* to colonise ruminant niches. Despite isolation from sheep milk, both strains of *S. equinus* produced less nisin E when cultured with lactose (Figure 5). Nisin A expression has been shown to be lactose/galactose inducible, in addition to the traditional NisRK induction [55]. This expression has been attributed to the presence of a NisRK regulated TCT-N8-TCT direct repeat upstream of the *nisA* promoter [56]. Expression by lactose is not apparent in *S. equinus* or in nisin P expression from *S. agalactiae* DPC7040 (Figure 5), which lack a similar repeat 54 bp upstream of the conserved promoter region (Supplementary Figure S2). However other non-lactococcal producers lack the repeat sequence and express nisin when cultured on lactose. This may suggest that the repeat sequence is not required for the expression of nisin on lactose, and that another mechanism limits nisin E and P production on the carbohydrate. 

Nisin A has a dual mechanism of action, wherein it binds lipid II, preventing cell wall biosynthesis, and subsequently forms pores in bacterial cell membranes. Nisin variants are typically broad spectrum in nature, inhibiting a range of Gram-positive species, but variants may possess different specific activities or be differentially produced. The recently described nisin G produced by *S. salivarius* DPC6487 was found to be selectively active against 9 of 23 tested bacteria (21 Gram-positive), including Gram negative *Fusobacterium* spp., whereas a nisin A producer inhibited all 21 Gram-positive isolates to varying degrees [27]. Similarly, nisin E inhibited 9 of the 40 Gram-positive bacteria screened, including *Bacillus firmis* DPC6349, *Clostridioides* spp., *Lactobacillus* spp., and *Staphylococcus intermedius* DSM20373 (Table 2). It remains to be determined if this is due to a higher minimum inhibitory concentration of nisin E, that it is poorly expressed relative to other nisin variants, or a combination of these factors. 

Nisin E is the second natural variant of 32 residues to be described, the first being the distantly related nisin O4, produced by the human gut bacterium, *Blautia obeum* [22]. Nisin E contains 10 differences from nisin A, including 8 substitutions, Ile4Lys, Gly18Thr, Asn20Pro, Met21Ile, His27Gly, Val32Phe, Ser33Gly, and Lys34Asn, and 2 deletions, Ser29 and Ile30 (Figure 2b). Nisin E is very similar to nisin U/U2 and P with just three (93% identity), and four (90% identity) amino acid differences, respectively. Specifically, Ala15, common to both nisin A and E, is changed into an Ile residing in nisin U, while Met21 is changed to Ile in nisin E and Leu in nisin U. The peptide is one amino acid longer than nisins U [23] and P, as nisin E contains an Asn32 residue which is absent in the other 31 amino acid peptides and all other nisins. *S. equinus*, *S. agalactiae, S. gallolyticus* subsp. *pasteurianus*, and *S. uberis* are closely related and frequently inhabit the same niches, facilitating gene transfer events that may explain the similarity between nisin’s E, P, and U. If streptococcal nisin variants are frequently encountered by bacteria in animal GI-tracts, then maintaining nisin production and immunity systems would also be beneficial for competition survival. Nisin H, produced by *S. hyotintestinalis* DPC6484, is less similar to nisins E, P, and U (sharing ~67–70% amino acid identity) and more similar to nisin A (85% identity), which may suggest a more recent divergence from lactococcal nisins, which is also reflected in gene cluster structure. Nisin E lacks a serine at position 29, which in nisin A, makes the peptide susceptible to cleavage and inactivation by the nisin resistance protein (Nsr). Therefore, nisin E could be a desirable natural variant that escapes Nsr peptide inactivation, though this has yet to be demonstrated. Nisin E also contains a proline at position 20 which, when bioengineered in nisin A, shows an increased specificity of the peptide towards *Staphylococcus aureus;* taken together these changes may indicate nisin as a useful natural nisin variant for therapeutic purposes. However, previous studies have demonstrated that shortening the C-terminus of nisin A to 31 residues reduces its activity 10 fold, but nisin^1–32^ exhibits similar activity to that of the full length peptide. Extending the C-terminus has been found to improve the permeation of cell membranes by the peptide and to increase activity against Gram-negative bacteria [57]. However, it remains to be determined if the additional Asn residue impacts the activity relative to other nisins, particularly as Asn is a polar amino acid.

Among the variants, the hinge region of natural nisin variants displays a large degree of amino acid variation in residues 20 and 21. The hinge region of nisin A (NMK) is conserved in lactococcal-derived nisins, with the exception of nisin Q (NLK), which contains one amino acid difference. The hinge region of nisin E (PIK) more closely resembles the streptococcal-derived hinge regions, i.e., Nisin U and O (PLK) and nisin P (AIK). This variation likely impacts activity, as bioengineering of the nisin A hinge region has been previously demonstrated to alter the activity of nisin variants [16]. The isoleucine at position 21, within the hinge region of the nisin E peptide (PIK), is also present in nisin P (AIK) [25,26], although the specific three-residue hinge combination is unique to nisin E (Figure 2b).

Nisin E genes were detected among *S. equinus* species and not in other *Streptococcus* spp., including closely related members of the SBSEC. As such, nisin E production may be a unique feature of the species *S. equinus,* whereas nisin P has been found to be produced by both *S. gallolyticus* ssp. *pasteurianus* and by *S. agalactiae* DPC7040 [25,26]. The nisin E gene cluster encodes all of the genes typically involved in nisin production, including transport, modification, and immunity. However, the gene order differs from that of other nisin gene clusters, with the structural peptide immediately upstream from the *lanFEG* transport and immunity genes (Figure 2a). The nisin U gene cluster possesses transposases flanking the gene cluster, as well as another directly upstream from *nsuA*, to which the reorganisation of the cluster relative to nisin A is attributed [23]. The gene rearrangement of the nisin U gene cluster relative to nisin A is also observed in the similar streptococcal nisin P gene cluster (Figure 2). No transposase sequences were found in the nisin E gene cluster to indicate an obvious mechanism of gene rearrangement.

We sought to predict the promoters present in the nisin E gene cluster through sequence alignment with previously characterised promoters in the *Lactococcus lactis* nisin A gene cluster to provide further insights on nisin expression (Figure 4). The nisin A gene cluster contains two constitutive promoters for the transcription of the nisin immunity protein (*nisI*) and the response-regulator histidine kinase two-component system (*nisRK*), respectively [29,58]. It also contains two inducible NisRK-regulated promoters responsible for the expression of the nisin core peptide production (*nisABTCI*) and transport/immunity genes (*nisFEG*)(Figure 4) [29]. The constitutive promoter upstream of *nisR* in *L. lactis* is not conserved in *Streptococcus* spp., although we identified putative −35/−10 regions potentially responsible for NisRK expression. *S. equinus* APC4007 and 4008 share a conserved −10 region and −35, which overlaps with a TCT-N8-TCT direct repeat that is highly conserved across all NisRK regulated promoters (P_nisA_ and P_nisF_). Two such repeats upstream of the transcription start site have been found to be optimal for inducible nisin expression in *Lactococcus lactis* [56]. Exploring the absence and presence of these repeats across nisin producers may be of interest in future expression studies to increase production of natural variants, some of which are known to be poorly expressed [22,25,26]. We also predict a third nisin-inducible promoter upstream of the serine peptidase gene (*nseP*) in the nisin E cluster, which is conserved in nisin U and P and would be essential for expression, given their location at the periphery of the gene cluster.

We predict a number of Rho-independent (stem loop) transcription terminators within the nisin E gene cluster, including the presence of a terminator within the *nseR* open reading frame that may result in reduced levels of nisin production, as has been previously demonstrated [59]. We note the similarity between predicted operon promoter and terminator structure (Figure 4) and the homology between peptide structures (Figure 3). Nisin J from *S. capitis* APC2923 does not cluster with other nisin variants, and indeed, the nucleotide sequences are highly divergent from other nisin production gene clusters. Interestingly, nisin H from *S. hyointestinalis* DPC6484 clusters more closely with lactococcal nisins, and the nucleotide sequences containing predicted promoters are dissimilar to nisin E, P, and U (Supplementary Figure S2). Nisin E clusters with closely related streptococcal variants nisin U and P, and is also more related to nisins O1,2,3, and O4 from *B. obeum* than its lactococcal variants.

Nisin E sensing and export/immunity genes (*nseRKFEG*), without corresponding production machinery genes, were found in 45.5% of publicly available *Streptococcus equinus* genomes (20/44) (Figure 7). Nisin resistance/immunity gene clusters have previously been described as consisting of an S41 peptidase nisin resistance protein (NSR) and a BceAB-type ABC transporter (NsrFP), which are regulated by the nisin response-regulator histidine-kinase two-component system (NisRK) [30,60]. Genes encoding NSR have been detected in a range of pathogenic and non-pathogenic bacteria, including *Corynebacterium* spp., *Leuconostoc* spp., *Enterococcus faecium*, *Staphylococcus* spp., and *Streptococcus* spp., and typically confer high levels of resistance to nisin [30,61]. The genes identified in *S. equinus* are distinct from the nisin resistance gene cluster and are homologous to nisin E genes, with 19/20 strains encoding proteins with greater than 90% amino acid identity to NseRKFEG encoded in *S. equinus* APC4007 and APC4008 (Supplementary Figure S5). Previous comparative genome analysis of 43 *L. lactis* genomes identified a subset of four strains retaining *nisFEG/nisI* genes, without biosynthesis genes, but did not determine if these strains retained full immune capacity to nisin [62]. A gene-trait matching study of 710 individual *L. lactis* strains identified 59 strains that encoded *nisRKFEG*, without other biosynthetic machinery, and found that *nisFEG* always co-occurred with *nisRK* and imparted the ability to survive and acidify milk in the presence a 1.5 µg·mL^−1^ level of nisin, but was not as effective as the presence of *nsr* [63]. The same study identified the presence of the nisin immunity protein-encoding gene *nisI* co-localised with *nisP* in 16/710 genomes, which conferred some degree of nisin resistance [63]. The *nisIP* sub-gene cluster was not detected among *S. equinus* genomes, which could result from the fact that the two genes are at opposing ends of the nisin E gene cluster, rather than co-localised, as they are in the nisin A gene cluster, which would more easily facilitate co-retention after the loss of other genes (Figure 2a). The presence of *nseRKFEG* in *S. equinus* genomes likely confers a level of resistance to nisin E, enabling strains to colonise the same niche as nisin E producers, but without expending the energy involved in nisin production.

Nisin A is the prototypical lantibiotic, and it has been extensively researched and utilised since its discovery. Novel variants and related peptides continue to be identified across a multitude of genera, many of which are of interest in the context of the current and growing global AMR crisis. The continued discovery of novel nisin variants highlights the ubiquity of nisin-associated genes across prokaryotic genera, suggesting a strong role in Gram-positive bacterial competition in microbiomes. Features of the nisin E gene cluster in *S. equinus* shed light on the complexity of nisin cluster structure and expression and highlight some gaps in the current knowledge regarding the regulation of nisin variant expression, despite a long history of nisin expression system exploitation [64]. Further investigation of variant regulatory elements could result in improved production and enable in-depth characterisation and utilisation of non-lactococcal variants. Taken together, the discovery of this new nisin variant in some *S. equinus* strains, along with the finding that other strains apparently possess immunity determinants which are under nisin control, suggests a central role for nisin in the competitive nature of the species.

## Figures and Tables

**Table 1:** Nisin-producing strains used in this study and their growth conditions.

Organism	Strain	Nisin Variant	Temp.	O_2_	Medium
*Lactococcus lactis* ssp. *lactis*	ATCC11454	A	30	Aerobic	M17, 0.5% glucose
*Lactococcus lactis*	NZ9800 pCI372-nisA	A	30	Aerobic	M17, 0.5% glucose, 10 µg·mL^−1^ Chloramphenicol
*Lactococcus lactis*	NZ9800 pCI372-nisZ	Z	30	Aerobic	M17, 0.5% glucose, 10 µg·mL^−1^ Chloramphenicol
*Lactococcus lactis*	NZ9800 pCI372-nisF	F	30	Aerobic	M17, 0.5% glucose, 10 µg·mL^−1^ Chloramphenicol
*Lactococcus lactis*	NZ9800 pCI372-nisQ	Q	30	Aerobic	M17, 0.5% glucose, 10 µg·mL^−1^ Chloramphenicol
*Staphylococcus capitis*	APC2923	J	37	Aerobic	BHI
*Streptococcus uberis*	42	U	37	Aerobic	BHI
*Streptococcus equinus*	APC4007	E	37	Aerobic	BHI
*Streptococcus equinus*	APC4008	E	37	Aerobic	BHI
*Streptococcus hyointestinalis*	DPC6484	H	37	Anaerobic	BHI
*Streptococcus agalactiae*	DPC7040	P	37	Aerobic	BHI

**Table 2:** Spectrum of inhibition of nisin E producers *S. equinus* APC4007 and APC4008 against bacterial strains, as determined by a deferred antagonism assay.

Organism	Strain	Temp	O_2_	Media	Inhibition	
					4007	4008
*Bacillus cereus*	NCIMB700577	37	Aerobic	BHI	−	−
*Bacillus subtilis*	S249	37	Aerobic	BHI	−	−
*Bacillus thuringiensis*	DPC6341	37	Aerobic	BHI	−	−
*Bacillus firmis*	DPC6349	37	Aerobic	BHI	+++	+++
*Clostridioides difficile*	DPC6534	37	Anaerobic	RCM	+	+
*Clostridioides sporogenes*	LMG10143	37	Anaerobic	RCM	+	+
*Enterococcus faecium*	NCDO0942	37	Aerobic	BHI	−	−
*Enterococcus faecium (VRE)*	APC1026	37	Aerobic	BHI	−	−
*Enterococcus faecium (VRE)*	APC1032	37	Aerobic	BHI	−	−
*Enterococcus faecium (VRE)*	APC1033	37	Aerobic	BHI	−	−
*Enterococcus faecium (VRE)*	APC1039	37	Aerobic	BHI	−	−
*Enterococcus faecium (VRE)*	APC1044	37	Aerobic	BHI	−	−
*Enterococcus faecium (VRE)*	APC1055	37	Aerobic	BHI	−	−
*Lactococcus lactis*	HP	30	Aerobic	GM17	+	+
Lactococcus lactis *	ATCC11454	30	Aerobic	GM17	−	−
*Lactobacillus delbrueckii* ssp. *bulgaricus*	LMG6901	37	Anaerobic	MRS	+++	+++
*Lactobacillus delbrueckii* ssp. *lactis*	DPC5387	37	Anaerobic	MRS	+++	+++
*Lactobacillus helveticus*	DPC5358	37	Anaerobic	MRS	+	+
*Ligilactobacillus salivarius*	DPC6502	37	Anaerobic	MRS	+	+
*Listeria innocua*	DPC1768	37	Aerobic	BHI	−	−
*Listeria monocytogenes*	DPC3572	37	Aerobic	BHI	−	−
*Listeria monocytogenes*	L028	37	Aerobic	BHI	−	−
*Staphylococcus aureus*	32679	37	Aerobic	BHI	−	−
*Staphylococcus aureus*	C5M	37	Aerobic	BHI	−	−
*Staphylococcus aureus*	47.9	37	Aerobic	BHI	−	−
*Staphylococcus aureus*	DPC5243	37	Aerobic	BHI	−	−
*Staphylococcus aureus*	DPC7673	37	Aerobic	BHI	−	−
*Staphylococcus aureus*	R693	37	Aerobic	BHI	−	−
*Staphylococcus aureus (MRSA)*	DPC5646	37	Aerobic	BHI	−	−
*Staphylococcus epidermidis*	DSM3095	37	Aerobic	BHI	−	−
*Staphylococcus intermedius*	DSM20373	37	Aerobic	BHI	+	+
*Streptococcus agalactiae*	35	37	Aerobic	BHI	−	−
*Streptococcus agalactiae*	119	37	Aerobic	BHI	−	−
*Streptococcus agalactiae*	APC1055	37	Aerobic	BHI	−	−
*Streptococcus agalactiae*	ATCC13813	37	Aerobic	BHI	−	−
*Streptococcus pneumoniae*	APC3850	37	Aerobic	BHI	−	−
*Streptococcus pneumoniae*	APC3857	37	Aerobic	BHI	−	−
*Streptococcus pyogenes*	DPC6992	37	Aerobic	BHI	−	−
*Streptococcus uberis*	ATCC5344	37	Aerobic	BHI	−	−
*Streptococcus uberis*	LL383	37	Aerobic	BHI	−	−

−, No activity; +, 0.5–1.5 mm inhibition zone; ++, 2–3.5 mm inhibition zone; +++, ≥4 mm inhibition zone; * nisin A producer.

## Data Availability

Genomic data from this study has been deposited to Genbank under accession numbers JANHMF000000000 and JANHME000000000.

## Supplementary Materials

The following supporting information can be downloaded at: https://www.mdpi.com/article/10.3390/microorganisms11020427/s1, Figure S1: Phylogram and genome average nucleotide identity (ANI) S. equinus APC4007 and S. equinus APC4008, Figure S2: Nucleotide sequence alignment of 200bp region upstream of nisA, nisF, nisP and homologs, Figure S3: Nucleotide sequence alignment of intergenic region upstream of nisR and homologous genes, Figure S4: Nucleotide sequence alignment of 400bp region upstream of nisI and homologous genes, Figure S5: Amino acid percent identity heatmaps of NseRKFEG protein; Table S1: Type and origin of samples from which strains were isolated, Table S2: Genomes of Streptococcus equinus used in pangenome analysis, Table S3: Nisin resistance protein sequences used in pan genome screen, Table S4: Nisin immunity protein sequences used in pan genome screen, Table S5: Nucleotide accessions containing nisin operon sequences, Table S6: ARNold predicted forward strand terminators from nisin variant operons, Table S7: Streptococcus equinus genomes encoding nse genes.
