# Supplementary material for: Nisin E Is a Novel Nisin Variant Produced by Multiple Streptococcus equinus Strains
Source: Microorganisms. 2023 Feb 8;11(2):427. doi: 10.3390/microorganisms11020427 (PMC9958725; doi:10.3390/microorganisms11020427)
Supplement: Supplementary file 1 [file microorganisms-11-00427-s001.zip › microorganisms-2163959-supplementary.pdf]

Supplementary Information

Supplementary Table S1. Type and origin of samples from which strains were isolated.

Sample Type	Number of Samples	Sample Source Location	Number of Isolates
Raw sheep's milk	74	New Zealand	293
Raw goat's milk	21	New Zealand	174
Raw cow's milk	9	Ireland	130
Other*	8	-	226

* Unpasteurised sheep, goat, cow and buffalo cheeses, fresh honey, honeycomb, sheep faeces, and canine saliva samples.

Supplementary Table S2. Genomes of *Streptococcus equinus* used in pangenome analysis.

Organism Scientific Name	Organism Qualifier	Assembly Name	Assembly Accession	Source	Level	Size
Streptococcus equinus	strain: ATCC 700338	ASM14640v1	GCF_000146405.1	RefSeq	Scaffold	2083084
Streptococcus equinus	strain: ATCC 9812	ASM18726v1	GCF_000187265.1	RefSeq	Scaffold	1741734
Streptococcus equinus	strain: SN033	ASM42142v1	GCF_000421425.1	RefSeq	Contig	1843423
Streptococcus equinus	strain: B315	ASM42404v1	GCF_000424045.1	RefSeq	Scaffold	1786673
Streptococcus equinus	strain: 2B	ASM70210v1	GCF_000702105.1	RefSeq	Scaffold	1919820
Streptococcus equinus	strain: HC5	ASM73108v1	GCF_000731085.1	RefSeq	Scaffold	1846241
Streptococcus equinus	strain: ATCC 33317	ASM74719v1	GCF_000747195.1	RefSeq	Contig	1841890
Streptococcus equinus	strain: JB1	Streptococcus bovis JB1	GCF_000747205.1	RefSeq	Contig	1954216
Streptococcus equinus	strain: AG46	ASM96431v1	GCF_000964315.1	RefSeq	Contig	1930741
Streptococcus equinus	strain: ICDDRB-NRC-S6	ASM148148v1	GCF_001481485.1	RefSeq	Contig	1849355
Streptococcus equinus	strain: FMD1	ASM452577v1	GCF_004525775.1	RefSeq	Contig	1935083
Streptococcus equinus	strain: NBRC 12057	ASM653894v1	GCF_006538945.1	RefSeq	Contig	1926856
Streptococcus equinus	strain: CNU 77-23	ASM975807v1	GCF_009758075.1	RefSeq	Complete Genome	1911874
Streptococcus equinus	strain: CNU G6	ASM975809v1	GCF_009758095.1	RefSeq	Complete Genome	1997064
Streptococcus equinus	strain: MDC1	ASM1404187v1	GCF_014041875.1	RefSeq	Complete Genome	1936555
Streptococcus equinus	strain: Colony399	ASM1690612v1	GCF_016906125.1	RefSeq	Chromosome	1904424
Streptococcus equinus	strain: C277	IMG-taxon 2623621021 annotated assembly	GCF_900099885.1	RefSeq	Scaffold	1825979
Streptococcus equinus	strain: GA-1	IMG-taxon 2593339267 annotated assembly	GCF_900100035.1	RefSeq	Contig	1799853
Streptococcus equinus	strain: MPR2	IMG-taxon 2651870307 annotated assembly	GCF_900100215.1	RefSeq	Scaffold	1863305

Streptococcus equinus strain: Sb05	IMG-taxon 2654588139 annotated assembly	GCF_900101445.1	RefSeq	Scaffold	1962621
Streptococcus equinus strain: Sb10	IMG-taxon 2654588134 annotated assembly	GCF_900101715.1	RefSeq	Contig	1887161
Streptococcus equinus strain: pGA-7	IMG-taxon 2608642168 annotated assembly	GCF_900102715.1	RefSeq	Scaffold	1797051
Streptococcus equinus strain: Sb09	IMG-taxon 2654588197 annotated assembly	GCF_900103355.1	RefSeq	Scaffold	2042259
Streptococcus equinus strain: Sb04	IMG-taxon 2651870306 annotated assembly	GCF_900104225.1	RefSeq	Contig	1875611
Streptococcus equinus strain: Sb17	IMG-taxon 2654588136 annotated assembly	GCF_900106895.1	RefSeq	Contig	1909978
Streptococcus equinus strain: Ye01	IMG-taxon 2654588209 annotated assembly	GCF_900109105.1	RefSeq	Contig	1931095
Streptococcus equinus strain: pR-5	IMG-taxon 2608642181 annotated assembly	GCF_900109395.1	RefSeq	Scaffold	1820620
Streptococcus equinus strain: H24	IMG-taxon 2654588151 annotated assembly	GCF_900109415.1	RefSeq	Scaffold	1875780
Streptococcus equinus strain: ES1	IMG-taxon 2623620585 annotated assembly	GCF_900110365.1	RefSeq	Scaffold	1827295
Streptococcus equinus strain: Sb18	IMG-taxon 2654588130 annotated assembly	GCF_900110845.1	RefSeq	Contig	1991056
Streptococcus equinus strain: MPR1	IMG-taxon 2651870309 annotated assembly	GCF_900110935.1	RefSeq	Scaffold	1864122
Streptococcus equinus strain: Sb13	IMG-taxon 2654588137 annotated assembly	GCF_900112385.1	RefSeq	Scaffold	1878198

Streptococcus equinus	strain: MPR4	IMG-taxon 2654588204 annotated assembly	GCF_900113235.1	RefSeq	Scaffold	1912748
Streptococcus equinus	strain: JB1	IMG-taxon 2593339156 annotated assembly	GCF_900114525.1	RefSeq	Scaffold	1967362
Streptococcus equinus	strain: SI	IMG-taxon 2654588207 annotated assembly	GCF_900115895.1	RefSeq	Scaffold	1912510
Streptococcus equinus	strain: Sb20	IMG-taxon 2654588135 annotated assembly	GCF_900115995.1	RefSeq	Scaffold	1894454
Streptococcus equinus	strain: AR3	IMG-taxon 2654588192 annotated assembly	GCF_900199575.1	RefSeq	Contig	2021307
Streptococcus equinus	strain: NCTC12969	42197_F02	GCF_900459295.1	RefSeq	Contig	1806069
Streptococcus equinus	strain: NCTC10386	42912_H01	GCF_900459945.1	RefSeq	Contig	1759508
Streptococcus equinus	strain: NCTC8140	42290_E02	GCF_900636465.1	RefSeq	Complete Genome	1873522
Streptococcus equinus	strain: NCTC10389	42880_B01	GCF_900636565.1	RefSeq	Complete Genome	1781995
Streptococcus equinus	strain: NCTC8133	42290_D02	GCF_901543475.1	RefSeq	Complete Genome	1867673
Streptococcus equinus	isolate: MGYG-HGUT-01308	MGYG-HGUT-01308	GCF_902373735.1	RefSeq	Scaffold	1925187
Streptococcus equinus	isolate: MGYG-HGUT-01363	MGYG-HGUT-01363	GCF_902374225.1	RefSeq	Scaffold	1741734

Supplementary Table S3. Nisin resistance protein sequences used in pan genome screen.

UniProt Entry	Entry Name	Protein names	Gene Names	Organism	Length
A0A0A0DLE3	A0A0A0DLE3_9STRE	Nisin-resistance protein	SSIN_0444	*Streptococcus sinensis*	314
A0A0G2Z1C9	A0A0G2Z1C9_STRAG	Nisin-resistance protein, putative	RDF_0967	*Streptococcus agalactiae*	117
A0A0G2Z5M0	A0A0G2Z5M0_STRAG	Nisin-resistance protein, putative	RDF_0966	*Streptococcus agalactiae*	179
A0A139P896	A0A139P896_9STRE	Nisin-resistance protein	STRDD11_00955	*Streptococcus sp. DD11*	315
A0A140UHB6	A0A140UHB6_STRAG	Putative nisin-resistance protein	SAL_1042	*Streptococcus agalactiae 515*	167
A0A1A9E115	A0A1A9E115_STRAG	Nisin-resistance protein, putative (Peptidase S41)	DK41_05165 NCTC8183_01371	*Streptococcus agalactiae*	320
A0A2X2M2N2	A0A2X2M2N2_STRAG	Nisin-resistance protein, putative (Peptidase S41)	D5F95_09920 NCTC8181_02301 NCTC8185_02307	*Streptococcus agalactiae*	320
A0A2X3VPC5	A0A2X3VPC5_STRSA	Nisin resistance protein	NCTC11085_01675	*Streptococcus sanguinis*	336
A0A2X3YJM2	A0A2X3YJM2_STRSA	Nisin resistance protein	NCTC11086_00744	*Streptococcus sanguinis*	336
A0A380IMG6	A0A380IMG6_STRAG	Nisin-resistance protein, putative	NCTC6175_01352	*Streptococcus agalactiae*	320
A0A380JWG9	A0A380JWG9_STRDY	Nisin-resistance protein	nsr NCTC4670_00790	*Streptococcus dysgalactiae subsp. dysgalactiae*	321
A0A380KH18	A0A380KH18_STREQ	Nisin-resistance protein	nsr NCTC11564_00976	*Streptococcus dysgalactiae subsp. equisimilis (Streptococcus equisimilis)*	321
A0A380L0L5	A0A380L0L5_9STRE	Nisin resistance protein	NCTC13765_01951	*Streptococcus massiliensis*	159
A0A380M477	A0A380M477_9STRE	Putative nisin-resistance protein	NCTC1080_00568	*Streptococcus viridans*	313
A0A448DKR6	A0A448DKR6_STREQ	Nisin-resistance protein	nsr NCTC6181_01847	*Streptococcus dysgalactiae subsp. equisimilis (Streptococcus equisimilis)*	321
A0A4U9YX70	A0A4U9YX70_STREQ	Nisin-resistance protein	nsr NCTC6407_00995	*Streptococcus dysgalactiae subsp. equisimilis (Streptococcus equisimilis)*	321
A0A4V0C1Q5	A0A4V0C1Q5_STRCB	Nisin-resistance protein	nsr NCTC6198_01136 TANIYAMA4_0743	*Streptococcus canis*	320

UniProt Entry	Entry Name	Protein names	Gene Names	Organism	Length
A0A4V0CQZ6	A0A4V0CQZ6_STRDY	Nisin-resistance protein	nsr NCTC7982_01632	*Streptococcus dysgalactiae*	321
A0A509DCT4	A0A509DCT4_9STRE	Nisin-resistance protein	nsr NCTC11567_01111	*Streptococcus sp. NCTC 11567*	321
A0A8B4IQF1	A0A8B4IQF1_STRUB	Nisin-resistance protein	nsr NCTC3858_01034	*Streptococcus uberis*	320
A0A8B6LB27	A0A8B6LB27_STREQ	Nisin-resistance protein (Peptidase S41)	nsr BBG08_07485 NCTC11557_01376	*Streptococcus dysgalactiae subsp. equisimilis (Streptococcus equisimilis)*	321
A3CPD7	A3CPD7_STRSV	Nisin resistance protein, putative	SSA_1656	*Streptococcus sanguinis (strain SK36)*	336
E6J086	E6J086_STRAP	Nisin-resistance protein	nsr HMPREF0813_006 56	*Streptococcus anginosus F0211*	317
F0FC69	F0FC69_STRSA	Nisin-resistance protein	nsr HMPREF9388_025 2	*Streptococcus sanguinis SK353*	336
F0FT01	F0FT01_STRSA	Nisin-resistance protein	nsr HMPREF9392_173 3	*Streptococcus sanguinis SK678*	336
F0I2W2	F0I2W2_STRSA	Nisin resistance protein	HMPREF9381_150 2	*Streptococcus sanguinis SK72*	336
F0I5L0	F0I5L0_STRSA	Nisin resistance protein	HMPREF9382_016 4	*Streptococcus sanguinis SK115*	314
F0IL53	F0IL53_STRSA	Nisin resistance protein	HMPREF9383_085 5	*Streptococcus sanguinis SK150*	336
F0IWA8	F0IWA8_STRSA	Nisin resistance protein	HMPREF9384_212 0	*Streptococcus sanguinis SK160*	336
F2BQW8	F2BQW8_STRSA	Nisin resistance protein	HMPREF9394_079 3	*Streptococcus sanguinis SK1057*	336
F2C524	F2C524_STRSA	Nisin resistance protein	HMPREF9386_017 7	*Streptococcus sanguinis SK330*	336
F2CCD0	F2CCD0_STRSA	Nisin resistance protein	HMPREF9391_015 8	*Streptococcus sanguinis SK408*	336
F3SGA9	F3SGA9_STRSA	Nisin resistance protein	HMPREF9397_018 1	*Streptococcus sanguinis SK1087*	336
F3U958	F3U958_STRSA	Nisin resistance protein	HMPREF9393_016 1	*Streptococcus sanguinis SK1056*	336
F3UTK7	F3UTK7_STRSA	Nisin resistance protein	HMPREF9389_216 5	*Streptococcus sanguinis SK355*	336
F3UW53	F3UW53_STRSA	Nisin resistance protein	HMPREF9380_074 1	*Streptococcus sanguinis SK49*	336
M4YXC9	M4YXC9_STREQ	Putative nisin-resistance protein	GGS_0959	*Streptococcus dysgalactiae subsp. equisimilis RE378*	321
Q8DZX2	Q8DZX2_STRA5	Nisin-resistance protein, putative	SAG0973	*Streptococcus agalactiae serotype V (strain ATCC BAA-611 / 2603 V/R)*	320

Supplementary Data S4. Nisin immunity protein sequences used in pan genome screen.

UniProt Entry	Entry Name	Protein Name	Gene Name	*Organism*	Length
P42708	NISI_LACLL	Nisin immunity protein	*nisI*	*Lactococcus lactis* subsp. *lactis* *(Streptococcus lactis)*	245 AA
Q2QBS6	Q2QBS6_STRUB	NsuI	*nsuI*	*Streptococcus uberis*	238 AA
A0A6C0GXF1	A0A6C0GXF1_9STRE	Immunity protein NspI	*nspI*	*Streptococcus gallolyticus*	238 AA
B5MEU0	B5MEU0	Immunity protein	*niqI*	*Lactococcus lactis*	238 AA
Q45403	Q45403_BACIU	SpaI	*spaI*	*Bacillus subtilis*	165 AA
A0A286N5V2	A0A286N5V2_9FIRM	NsoI	*nsoI*	*Blautia obeum*	278 AA
-	-	NseI	*nseI*	*Streptococcus equinus*	245 AA

Supplementary Table S5. Nucleotide accessions containing nisin operon sequences

Species	Strain	Nisin Variant	NCBI Genome Accession	Assembly level
Lactococcus lactis subsp. *lactis*	ATCC11454	A	*	Contig
Lactococcus lactis subsp. *lactis*	IO-1	Z	AP012281	Complete genome
Lactococcus lactis	61-64	Q	AB362350	Contig
Streptococcus hyointestinalis	DPC6484	H	KP793707	Contig
Streptococcus agalactiae	DPC7040	P	WIDP01000009	Contig
Streptococcus uberis	42	U	DQ146939	Contig
Staphylococcus capitis	APC2923	J	MN602039	Contig
Blautia obeum	A2-162	O	KY914474	Contig
Streptococcus equinus	APC4007	E	JANHMF000000000	Contig
Streptococcus equinus	APC4008	E	JANHME000000000	Contig
*Accessed from genomes.atcc.org/				

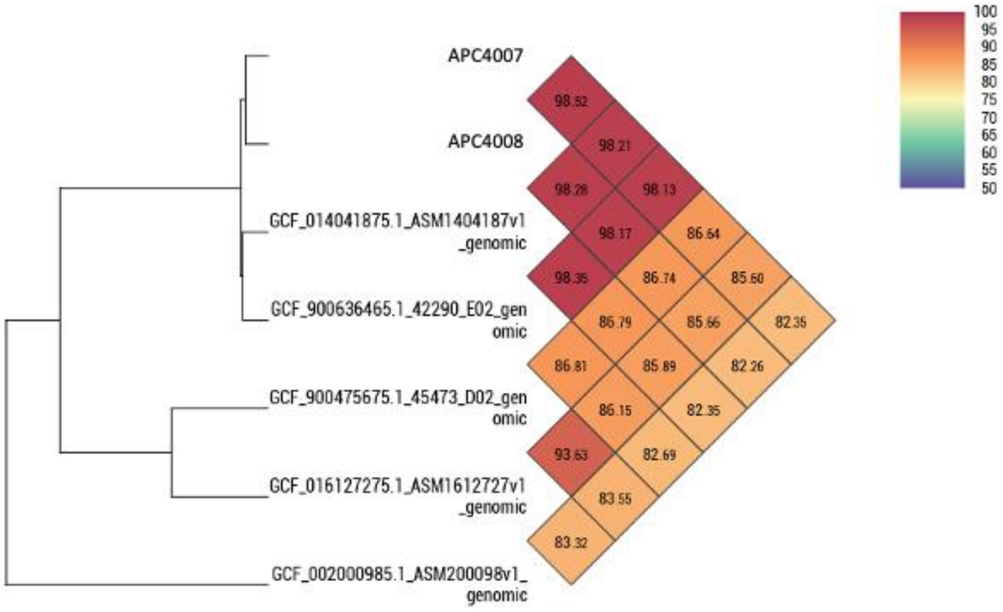

Supplementary Figure S1. Phylogram and genome average nucleotide identity (ANI) *S. equinus* APC4007 and *S. equinus* APC4008 compared with related *Streptococcus* spp. of the SBSEC. ANI values were calculated and visualised using OrthoANI[1].

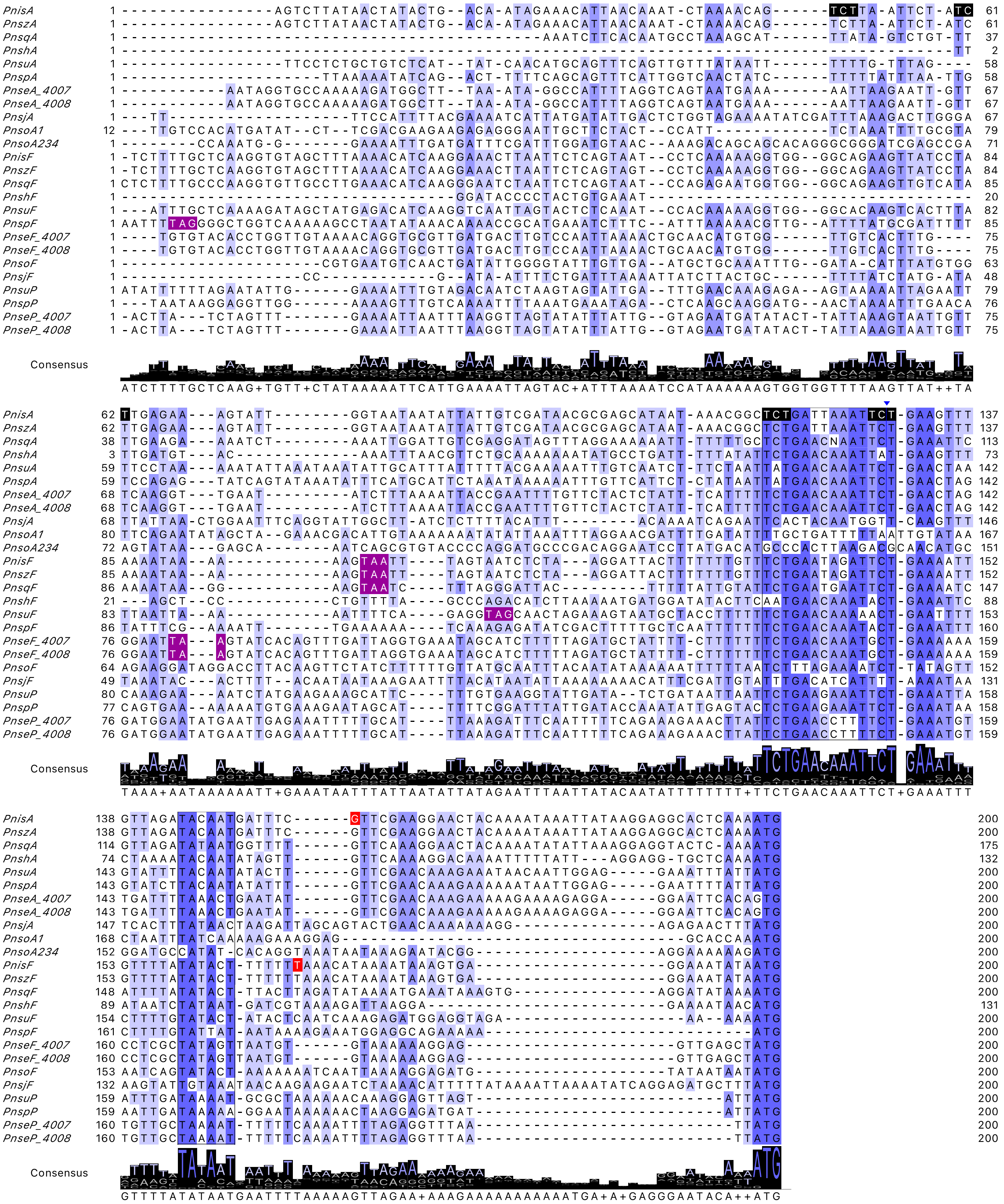

Supplementary Figure S2. Nucleotide sequence alignment of 200bp region upstream of nisA, nisF, nisP and homologs. Promoter -10 TATAAT and conserved -35 TCT-N8-TCT sequences are boxed. Nucleotides coloured blue by conservation. Stop codons of genes upstream are highlighted in burgundy. The TCT-N8-TCT at -107 in *Lactococcus lactis* ssp. *lactis* is highlighted in black. Previously identified transcription start sites (+1) are highlighted in red.

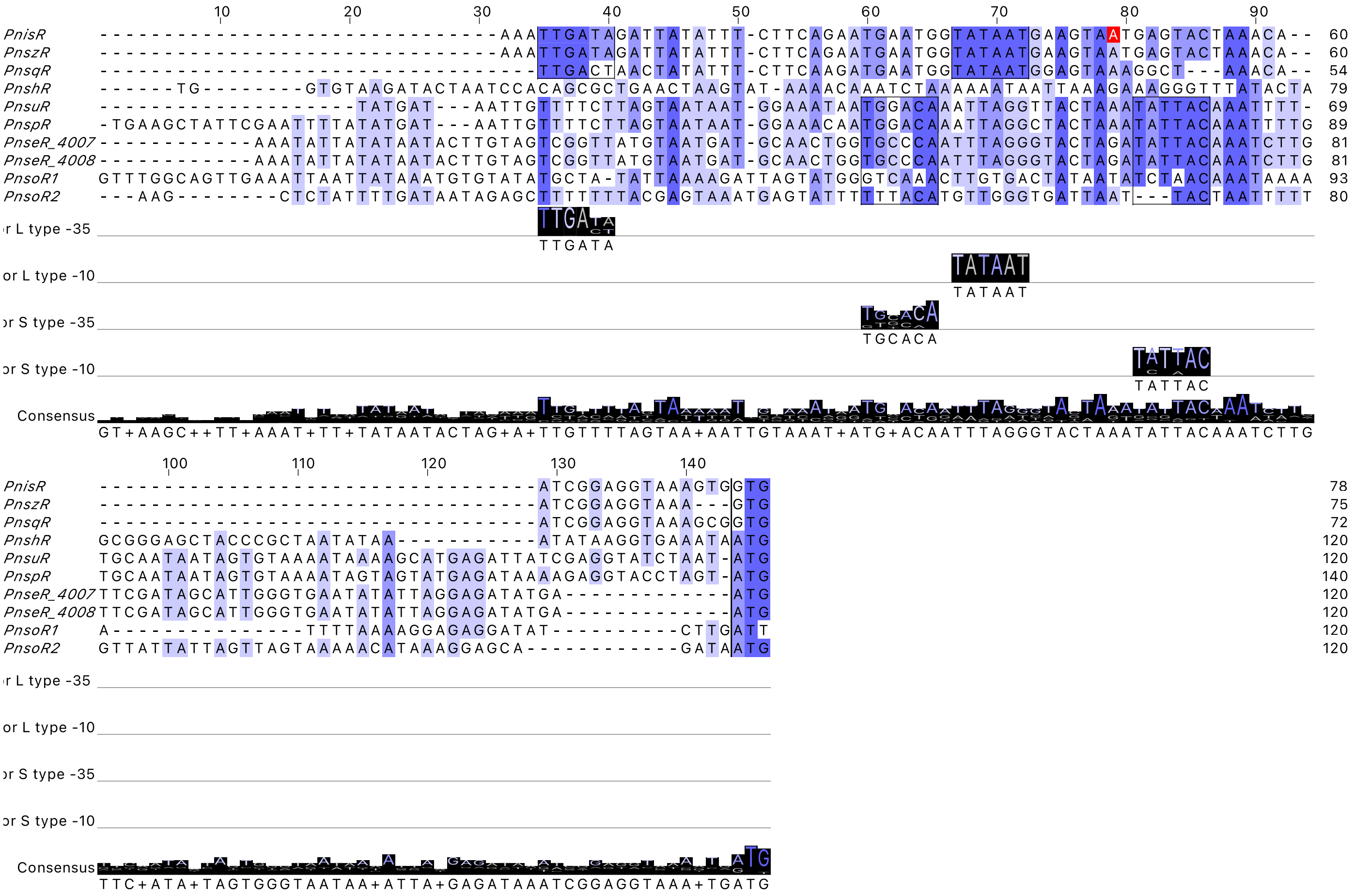

Supplementary Figure S3. Nucleotide sequence alignment of intergenic region upstream of nisR and homologous genes. Putative *Lactococcus* (L type) and *Streptococcus* (S type)-35 and -10 signals, ribosome binding sites and start codons are boxed with consensus sequences displayed below the alignment. Previously identified transcription start site (+1) highlighted in red. Nucleotides coloured by conservation.

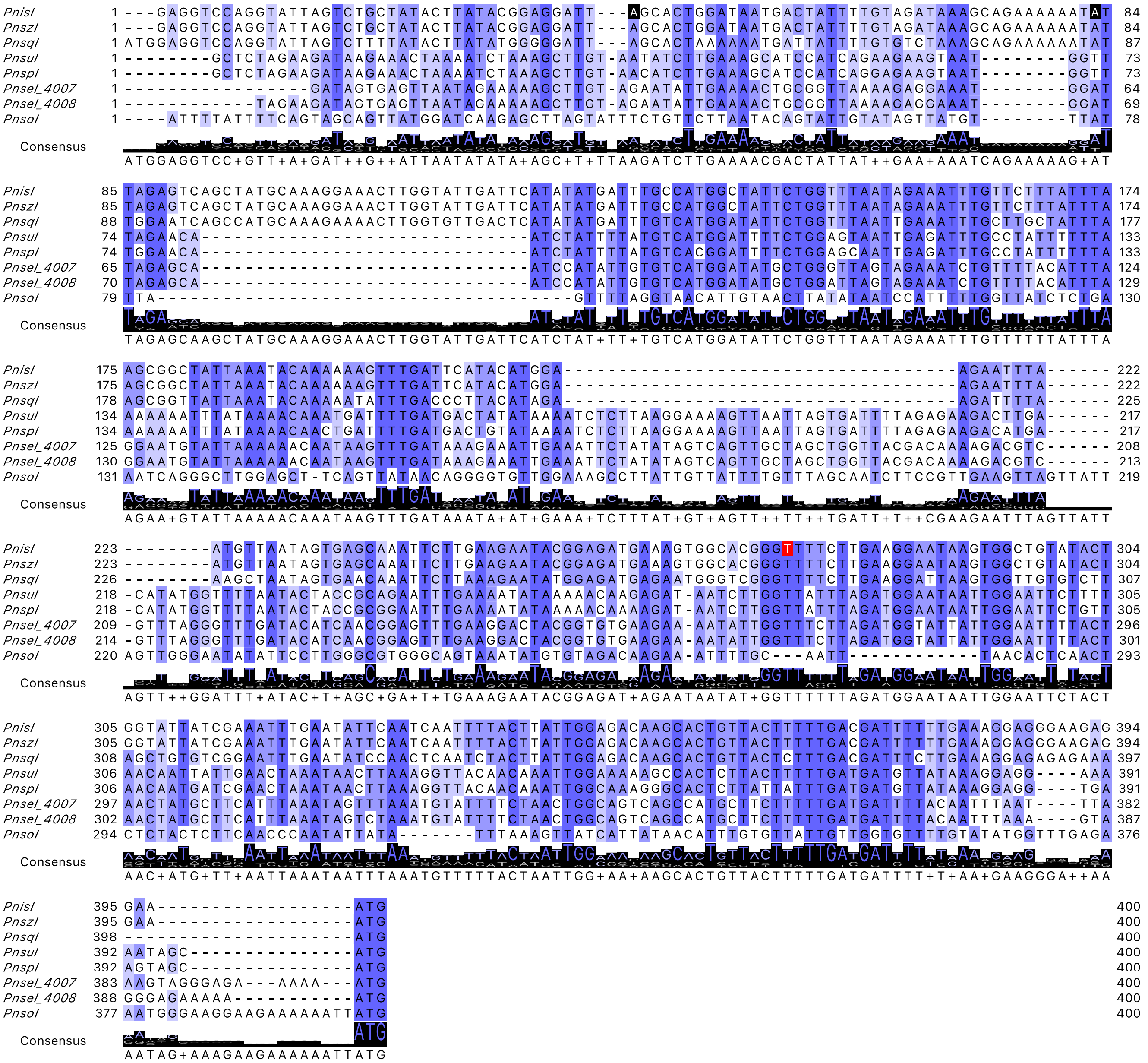

Supplementary Figure S4. Nucleotide sequence alignment of 400bp region upstream of nisI and homologous genes. Nucleotides coloured by conservation. Previously identified transcription start site (+1) highlighted in red. -234 and -194 nucleotides are highlighted in black.

Supplementary Table S6. ARNold predicted forward strand terminators from nisin variant operons.

Nisin A

Total number of predicted transcription terminators: 8

173 Both + AACCAAATCAAAGGATAGTATTTTGTTAGTTCAGACATGGATACTATCCTaTTTTTATAAGTTA -7.90

3773 Rnamotif + CATGGAACATTGGGGTAGCAATTTTACTCCTTATTGTTCCAG -6.70

11173 Both + AAAAATAAAAAAGTAATTTAGTAATCTCTAAGGATTACTTTTTTTGTTTCTG -3.40

13661 Erpin + ACAATGAAAAATCCAAGTATAAATACTTGGATTTTTCATTATTT -8.90

Nisin Z

Total number of predicted transcription terminators: 8

173 Both + AACCAAATCAAAGGATAGTATTTTGTTAGTTCAGACATGGATACTATCCTaTTTTTATAAGTTA -7.90

3773 Rnamotif + CATGGAACATTGGGGTAGCAATTTTACTCCTTATTGTTCCAG -6.70

11173 Both + AAAAATAAAAAAGTAATTTAGTAATCTCTAAGGATTACTTTTTTTGTTTCTG -3.40

13661 Erpin + ACAATGAAAAATCCAAGTATAAATACTTGGATTTTTCATTATTT -8.90

Nisin Q

Total number of predicted transcription terminators: 2

344 Both + AACCAAATCAAAGGATAGTATTTTGTTGACTTAGGTAAAGATACTATCCTaTTTTTATAAGTTA -7.90

3943 Rnamotif + CATGGAACATTGGAGTAGCATTTTTACTCCTTATTGTTCCAA -7.60

Nisin U

Total number of predicted transcription terminators: 7

1591 Rnamotif + TCCATTTCAACTCCTAAAGTCTCTGCAGCTTTGGGATTGATTATAGAA -7.70

3810 Erpin + AAAATTTTCAGAGGTAGCAACTAGAAAGTAATGCTACCTTTTTTCTGAACAA -10.30

6053 Both + ACAAGCAAAACACTTCTGTTGAAATCTAGTAAACTAGAAACAGGAGTaTTTTGTTATGGTC -6.80

7070 Rnamotif + GTTAAAATAAAGGTAGTGGATAGATAGCTTGCTACCTTTATTTTTATA -6.70

13995 Both + AATTTAGAACAGCAAGTTCAGGAGAGTTTGCTATTTTTTGTCA -5.60

14922 Erpin + TTCATACAAACTGCCCTCCTATTATTAGATTTTATGTCTAACAATAGGAGGGCAgTTCAAAGTGAATT -13.90

Nisin P

Total number of predicted transcription terminators: 5

1263 Both + CAATTTCGACTCCTAAAGTCTCTGCAGCTTTAGGaTTGATTATAGAA -8.80

3514 Erpin + ATAAACAAAACCGCATGAAATCTTTCATTTAAAAACGTTGATTTTATGCGaTTTTTTATTTCGA -6.80

6429 Both + GCTAAAATAAAGGTAGTGGATAGTAGTCTACTACCTTTATTTTTATA -11.20

7044 Rnamotif + AGTTCACTAAAGTTTATCAACTTATTGATGAGCTTTGTTGTGATA -7.10

13361 Both + AAAATATAGATCAGCAAGTTCAGAAGAGCTTGCTGTTTTTTGGTACA -10.60

Nisin E

Total number of predicted transcription terminators: 11

2184 Both + TATTCCGTTAACCAGCAGAGAATATGATTTGCTGGaTTTGCTATCAGC -11.70

3821 Both + AATTATATGAAGAGGTTGAGTTTAAAAGTGTTCAACCTCtTTGTTTATTTTGC -10.20

3963 Both + TGCCAAAAAGATGGCTTTAAATAGGCCATTTTAGGTCAGT -6.80

4338 Both + ATTAGGTGAAATAGCATCTTTTTAGATGCTATTTTCTTTTTTTC -9.90

4589 Rnamotif + TTAGAAGAAGCTGACAGTGGTGCTGTTGTTTTTAATGGTG -4.30

5020 Rnamotif + TGCTGACCACATTGTCATTTTACATGATGGCGAaaTTTTTTATGATG -7.00

5264 Rnamotif + TTCGGTTTATTGGTGGGAAGCTATCTTTTTGTACACT -3.80

6618 Rnamotif + AAGTTGGAAAAGGTAGTATATAAAATAGCTACCTTTATTTTTATA -6.40

13497 Both + AATTAAATCAACCAGACAATCTATTTTTGTCTGGgTTTTATTTGTCA -11.90

Nisin O

Total number of predicted transcription terminators: 6

635 Rnamotif + ATGTGGAGAAGGATAGGACCTTACAAGTTCTATCTTTTTTGTTATG -7.90

5587 Rnamotif + CCGTAGATCATTCTCCTACACATGGGGAGATTATTTTTAACG -7.20

5871 Rnamotif + GTTCAGGTTAGCTCTGGTGGAATACACCAGAGTTTTTTACTTT -14.20

5913 Both + TTATAGGATTAGGGTGTCAAAAGCCCaTTTTAATATTTG -5.50

7132 Erpin + ATACATAAAAAAGCTCTATTTGATAATAGAGCTTTTTTACGAGTA -8.90

Nisin J

Total number of predicted transcription terminators: 2

2136 Rnamotif + TTATTTGTAATTTTATTGTTGATAGCAGTAGATATTATTTTAAT -4.60

6650 Rnamotif + TACTTTTAGACGAGCCTAGTTCGGCTCTTGATTTAGAAT -8.60

Supplementary Table S7. *Streptococcus equinus* genomes encoding *nse* genes.

Strain	Recorded Source	Genome Assembly Accession	Encodes
APC4007	Sheep milk		*nsePRKAFEGBTCI*
APC4008	Sheep milk		*nsePRKAFEGBTCI*
SI	-	GCA_900115895.1	*nsePRKAFEGBTCI*
MDC1	Canine oral cavity	GCA_014041875.1	*nsePRKAFEGBTCI*
GA-1	-	GCA_900100035.1	*nsePRKAFEGBTCI*
SN033 G637	-	GCA_000421425.1	*nsePRKAFEGBTCI*
B315 G597	-	GCA_000424045.1	*nsePRKAFEGBTCI*
pR-5	-	GCA_900109395.1	*nsePRKAFEGBTCI*
AG46	Sheep rumen	GCA_000964315.1	*nseRKFEG*
AR3	-	GCA_900199575.1	*nseRKFEG*
ATCC33317	Cow dung	GCA_000747195.1	*nseRKFEG*
C277	-	GCA_900099885.1	*nseRKFEG*
CNU 77-23	*Bos taurus* rumen fluid	GCA_009758075.1	*nseRKFEG*
CNU G6	*Bos taurus* coreanae rumen fluid	GCA_009758095.1	*nseRKFEG*
ES1	-	GCA_900110365.1	*nseRKFEG*
FMD1	Forest musk deer	GCA_004525775.1	*nseRKFEG*
H24	-	GCA_900109415.1	*nseRKFEG*
HC5	Cow rumen	GCA_000731085.1	*nseRKFEG*
MPR1	-	GCA_900110935.1	*nseRKFEG*
MPR2	-	GCA_900100215.1	*nseRKFEG*
MPR4	-	GCA_900113235.1	*nseRKFEG*
NBRC12057	Bovine rumen contents	GCA_006538945.1	*nseRKFEG*
NCTC8133	Infant faeces	GCA_901543475.1	*nseRKFEG*
NCTC8140	-	GCA_900636465.1	*nseRKFEG*
pGA-7	-	GCA_900102715.1	*nseRKFEG*
Sb04	-	GCA_900104225.1	*nseRKFEG*
Sb09	-	GCA_900103355.1	*nseRKFEG*
Ye01	-	GCA_900109105.1	*nseRKFEG*

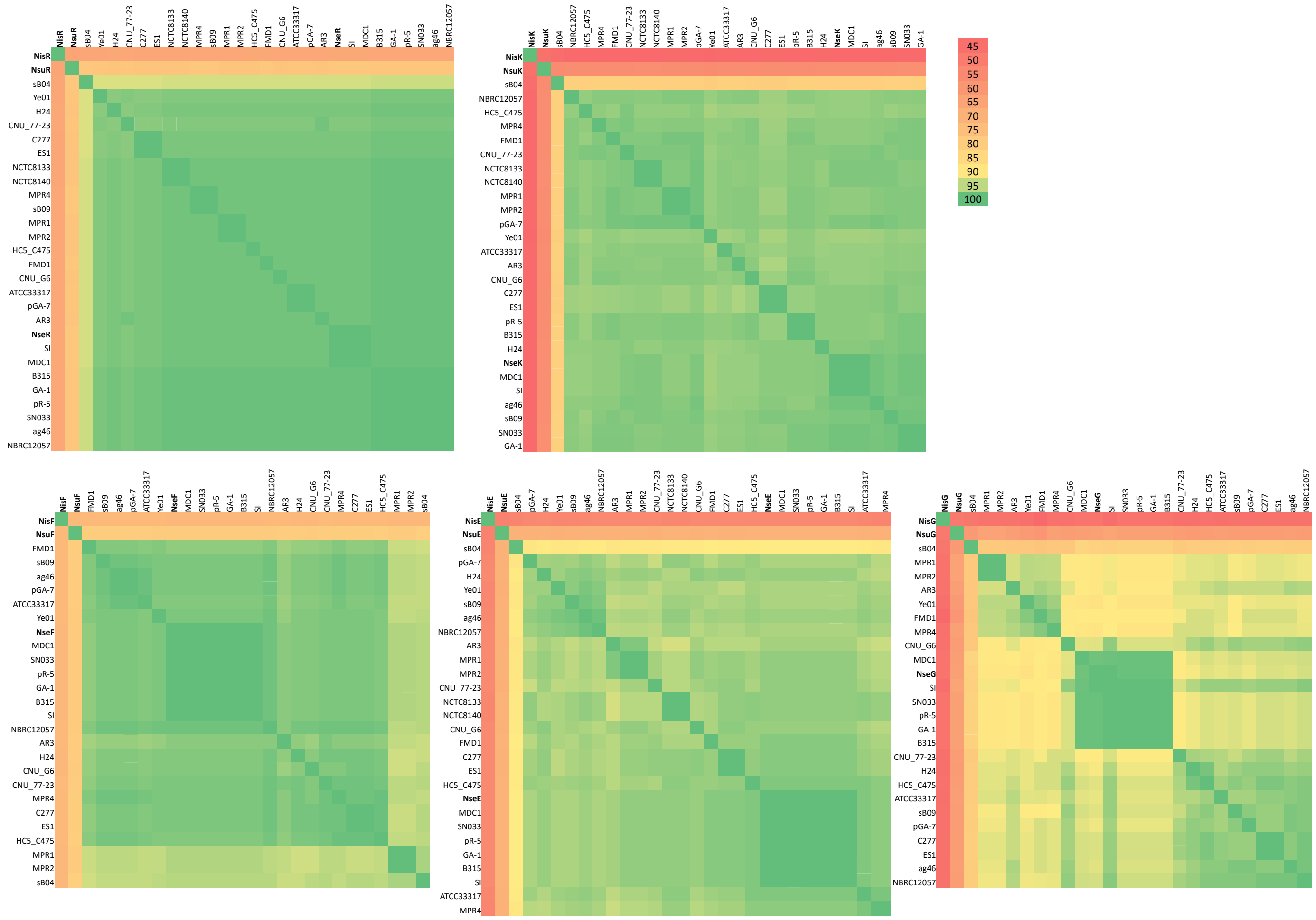

Supplementary Figure S5. Amino acid percent identity heatmaps of NseRKFEG proteins encoded in *S. equinus* genomes compared with *S. uberis* 42 nisin U (NsuRKFEG), and *Lactococcus lactis* ssp. *lactis* ATCC11454 nisin A (NisRKFEG) sequences.
